# Axo-glial interactions between midbrain dopamine neurons and oligodendrocyte lineage cells in the anterior corpus callosum

**DOI:** 10.1007/s00429-023-02695-y

**Published:** 2023-09-05

**Authors:** Megan Caldwell, Vanessa Ayo-Jibunoh, Josue Criollo Mendoza, Katherine R. Brimblecombe, Lauren M. Reynolds, Xin Yan Zhu Jiang, Colin Alarcon, Elizabeth Fiore, Jacquelyn N. Tomaio, Greg R. Phillips, Susana Mingote, Cecilia Flores, Patrizia Casaccia, Jia Liu, Stephanie J. Cragg, Dan P. McCloskey, Leora Yetnikoff

**Affiliations:** 1 CUNY Neuroscience Collaborative, The Graduate Center, City University of New York, 365 5Th Ave, New York, NY 10016 USA; 2 Department of Psychology, College of Staten Island, City University of New York, 2800 Victory Boulevard, Staten Island, NY 10314 USA; 3 Department of Biology, College of Staten Island, City University of New York, 2800 Victory Boulevard, Staten Island, NY 10314 USA; 4 Centre for Integrative Neuroscience, Department of Physiology, Anatomy and Genetics, University of Oxford, Oxford, OX1 3PT UK; 5 Oxford Parkinson’s Disease Centre, University of Oxford, Oxford, OX1 3PT UK; 6 Aligning Science Across Parkinson’s (ASAP) Collaborative Research Network, Chevy Chase, MD 20815 USA; 7 Plasticité du Cerveau, CNRS UMR8249, École Supérieure de Physique et de Chimie Industrielles de la Ville de Paris (ESPCI Paris), Paris, France; 8 Neuroscience Initiative, Advanced Science Research Center, Graduate Center of The City University of New York, New York, NY USA; 9 Center for Developmental Neuroscience, College of Staten Island, City University of New York, 2800 Victory Boulevard, Staten Island, NY 10314 USA; 10 Department of Psychiatry and of Neurology and Neuroscience, McGill University, and Douglas Mental Health University Institute, Montreal, QC Canada; 11 Department of Neuroscience and Neurology, Icahn School of Medicine at Mount Sinai, New York, NY 10029 USA

**Keywords:** Dopamine-glial interactions, Dopamine d1 receptor, Dopamine d2 receptor, Oligodendrocyte progenitor cells, Myelin plasticity

## Abstract

Oligodendrocyte progenitor cells (OPCs) receive synaptic innervation from glutamatergic and GABAergic axons and can be dynamically regulated by neural activity, resulting in activity-dependent changes in patterns of axon myelination. However, it remains unclear to what extent other types of neurons may innervate OPCs. Here, we provide evidence implicating midbrain dopamine neurons in the innervation of oligodendrocyte lineage cells in the anterior corpus callosum and nearby white matter tracts of male and female adult mice. Dopaminergic axon terminals were identified in the corpus callosum of DAT-Cre mice after injection of an eYFP reporter virus into the midbrain. Furthermore, fast-scan cyclic voltammetry revealed monoaminergic transients in the anterior corpus callosum, consistent with the anatomical findings. Using RNAscope, we further demonstrate that ~ 40% of *Olig2* + */Pdfgra* + cells and ~ 20% of *Olig2* + */Pdgfra-* cells in the anterior corpus callosum express *Drd1* and *Drd2* transcripts. These results suggest that oligodendrocyte lineage cells may respond to dopamine released from midbrain dopamine axons, which could affect myelination. Together, this work broadens our understanding of neuron-glia interactions with important implications for myelin plasticity by identifying midbrain dopamine axons as a potential regulator of corpus callosal oligodendrocyte lineage cells.

**Supplementary Information:**

The online version contains supplementary material available at 10.1007/s00429-023-02695-y.

## Introduction

The impairment of neural activity-induced myelin regulation in the adult brain leads to social avoidance, motor learning deficits, and perturbations in the long-term retention of emotional memories, and it has been posited that myelin dysregulation may contribute to neuropsychiatric disorders (Ren et al. 2013). Understanding the neuronal cell types that communicate with oligodendroglia cells could therefore provide insight into the pathogenesis of neuropsychiatric disease and identify new possibilities for therapeutic intervention. Previous evidence has demonstrated roles for glutamate, GABA, and dynorphin in myelin regulation (Habermacher et al. 2019; Osso et al. 2021), and in the latter case, it was shown that dynorphin is released by unmyelinated axons to regulate the myelination of neighboring large-diameter axons.

Several lines of data implicate midbrain dopamine neurons in myelin regulation. Dopamine-related neuropsychiatric disorders, including schizophrenia and addiction, are associated with dysregulation of white matter tracts (Hampton et al. 2019; Karlsgodt 2016; Koshiyama et al. 2018; Lee et al. 2013; Lim et al. 2008; Ma et al. 2009; Mighdoll et al. 2015; Moeller et al. 2005; Rotarska-Jagiela et al. 2008; Samartzis et al. 2014; Tamnes and Agartz 2016; Xu and Li 2011). Reduced white matter integrity as measured by fractional anisotropy and decreased expression of myelin-associated genes have been reported, suggesting altered function of OPCs and oligodendrocytes in these clinical populations (Albertson et al. 2004; Kerns et al. 2010; Takahashi et al. 2011). In parallel, preclinical studies have demonstrated that, in contrast to their wild-type counterparts, dopamine D2 receptor knock-out mice fail to exhibit myelin regulation in response to chronic stress (Choi et al. 2017). While it is not clear from these findings whether white matter abnormalities are directly caused by altered dopamine neurotransmission, there is evidence that putative glial cells in the corpus callosum express D2 receptor mRNA (Howard et al. 1998) and that atypical antipsychotics, which act primarily through D2 receptors, enhance myelin repair following cuprizone-induced demyelination (Chandran et al. 2012; Templeton et al. 2019; Xu et al. 2010; Zhang et al. 2008, 2012; Zhornitsky et al. 2013). Furthermore, single-cell RNA sequencing of whole brain or cortical tissue demonstrates dopamine D1 and D2 receptor mRNA expression by oligodendrocyte lineage cells (Marques et al. 2016; Zhang et al. 2014).

The purpose of this study was to investigate whether midbrain dopamine neurons are positioned to make axo-glial connections in the anterior corpus callosum and nearby white matter tracts. The corpus callosum, known for being the largest white matter tract of the brain, contains an estimated ~ 0.5 to > 5 million axons (Walhovd et al. 2014), of which only 30% of axons are myelinated or partially myelinated (Sturrock 1980), providing a landscape ripe with opportunity for myelin plasticity. Indeed, glutamatergic synapses between axons and OPCs in the corpus callosum have been identified at the ultrastructural level (Kukley et al. 2007; Ziskin et al. 2007) and learning to perform a complex motor task increases OPC proliferation and differentiation and axon myelination in this region in a time-dependent manner (McKenzie et al. 2014; Marques et al. 2016; Xiao et al. 2016), while preventing this plasticity prevents motor learning. Similarly, optogenetic stimulation of primary motor (Gibson et al. 2014) or anterior cingulate (Piscopo et al. 2018) cortex neurons using frequencies that mimic neuronal activity upregulates OPC proliferation and differentiation and increases myelination in the callosal pathway. Intriguingly, dopamine receptor agonists and antagonists modulate myelination in vitro and in vivo, where agonists decrease differentiation of OPCs and antagonists increase OPC proliferation and stimulate oligodendrocyte differentiation (Bongarzone et al. 1998; Howard et al. 1998; Mi et al. 2018; Rosin et al. 2005; Wang et al. 2010).

For midbrain dopamine neurons to play a direct role in myelin plasticity of the corpus callosum, we would expect functional dopamine axons to be present in this white matter tract and that corpus callosal oligodendrocyte lineage cells express dopamine receptors. However, this has not yet been demonstrated. Here, we examine these questions using immunofluorescence, viral-mediated tract-tracing, voltammetry, and RNAscope in situ hybridization. We demonstrate that midbrain dopamine axons innervate the anterior corpus callosum, that these axons are functional, and that dopamine D1 and D2 receptor transcripts are expressed by oligodendrocyte lineage cells in this region.

## Materials and methods

This study used methods that are the same or similar to those in our previous work (Brimblecombe and Cragg 2015; Chang et al. 2021; Lopes et al 2019; Reynolds et al. 2015, 2019; Threlfell et al. 2021). As such, some text from the methods described herein are similar to those provided in those publications.

### Animals

All experiments and procedures were performed in accordance with the guidelines of the Institutional Animal Care and Use Committees of the College of Staten Island and Advanced Science Research Center, City University of New York; the Canadian Council of Animal Care and the McGill University/Douglas Mental Health University Institute Animal Care Committee; and the United Kingdom Animals (Scientific Procedures) Act 1986 and approved by the local ethical review panel at the Department of Physiology, Anatomy and Genetics, University of Oxford.

Wild-type (WT) C57/BL6 mice were bred in the Animal Facilities of the College of Staten Island and Advanced Science Research Center, City University of New York, and University of Oxford. PDGFRa^EGFP^ mice were bred at the College of Staten Island and DAT^cre^ mice were bred at the Douglas Mental Health University Institute Neurophenotyping Center. All mice were maintained on a 12-h light–dark cycle (light on at 0700 h) and given ad libitum access to food and water. Pups were weaned at postnatal day (PND) 21 ± 1 and housed with same-sex littermates.

### Surgeries

Adult (postnatal day (PND) 120 ± 15) male DAT^cre^ mice (*n* = 3) received unilateral stereotaxic infusions of the cre-dependent fluorophore virus DIO-eYFP (AAV-EF1a-DIO-EYFP; UNC Vector Core) into the VTA/SN. The following coordinates were used: − 3.2 mm (anterior/posterior), + 1.0 mm (lateral), and − 4.6 mm (dorsal/ventral) relative to Bregma, and at a 10° angle. A total of 0.5 μL of purified virus was delivered on each side over an 8-min period followed by a pause of 6 min. As part of a previous study (Reynolds et al. 2019), these mice received five intraperitoneal (i.p.) saline injections on alternate days from PND 75 ± 15 to PND 84 ± 15.

### Perfusions and immunofluorescence

Adult WT (PND 90 ± 15, *n* = 4; 2 male and 2 female) and PDGFRa^EGFP^ (PND 60 ± 15, *n* = 2; 1 male and 1 female) mice received an i.p. injection of ketamine/xylazine (> 75 mg/kg, i.p.) and were perfused intracardially with 50 ml of 0.9% chilled saline followed by 75 ml of chilled 4% paraformaldehyde in phosphate-buffered saline. Brains were dissected and post-fixed overnight in the same fixative solution at 4 °C. Brains were then transferred to phosphate-buffered saline until sectioned using a vibratome (50-μm-thick slices).

Adult DAT^cre^ mice received an overdose of sodium pentobarbital (> 75 mg/kg, i.p.) four to five weeks after virus injections and were perfused intracardially as described above. Brains were sectioned using a vibratome into 35-μm-thick slices. Alternate sections taken as part of a previously published experiment (Reynolds et al. 2019) were stored at – 20 °C in a cryoprotective solution before immunofluorescence processing for the current experiment.

Brain sections were processed for immunofluorescence according to standard protocols. Briefly, sections were rinsed in TBS solution (3 × 5 min), followed by Tris A and Tris B solutions (10 min each), followed by 2 h in blocking buffer (10% Normal Goat Serum and 0.1% Triton X in Tris B) at room temperature (RT). Primary antibodies (Rabbit GFP 1:1000 #ab290, Abcam; Chicken GFP 1:4000 #A10262, Thermofisher; Rabbit TH 1:1000 #OPA1-04050, Thermofisher; Rat MBP 1:100 #MAB386, Sigma-Aldrich) were diluted in Tris B and sections incubated at 4 °C for two–three nights. Sections were subsequently rinsed in Tris A and Tris B solutions (10 min each) and incubated in secondary antibodies (goat anti-rabbit Alexa Fluor 488, goat anti-rabbit Alexa Fluor 647, goat anti-chicken Alexa Fluor 647, and goat anti-rat Alexa Fluor 488 or 647) diluted 1:200 in Tris B for 2-h at RT followed by rinses in Tris A and Tris B solutions (10 min each). Finally, sections were mounted and coverslipped using ProLong diamond antifade mountant with DAPI (Invitrogen #P36961).

### RNAscope fluorescence in situ hybridization

Coronal 50-μm-thick brain sections from male and female WT mice were pre-mounted onto slides prior to RNAScope. An in situ hybridization screen for house-keeping genes was performed using RNAScope probes Rn-Polr2a (312481-C3), Rn-Ppib (313921-C3), Rn-Ubc (312011-C2) and reagents from Advanced Cell Diagnostics according to the manufacturer’s instructions. In situ hybridization was then performed using Olig 2 (447091-C2), Pdgfra (480661-C3), dopamine d1 (461901), and dopamine d2 (406501) probes. Briefly, sections were first fixed in chilled 4% paraformaldehyde for 15 min at 4 °C, dehydrated in increasing gradients of ethanol baths and left to air dry for 5 min. Endogenous peroxidase activity was quenched with hydrogen peroxide reagent for 10 min, followed by antigen retrieval for 5 min in boiling buffer. Immunohistochemistry was performed afterward for detection of Olig2, Pdgfra, dopamine d1, and dopamine d2 mRNA followed by protease digestion for 30 min at 40 °C. The RNAScope probes were then hybridized for 2 h at 40 °C in a humidity-controlled oven (HybEZ II, ACDbio, Newark,CA, USA). Successive addition of amplifiers was performed using the proprietary AMP reagents, and the signal was visualized through probe-specific horseradish-peroxidase-based detection by signal amplification with Opal dyes (Opal 520, Opal 570, Opal 690 Perkin Elmer, Waltham, MA, USA) diluted 1:1500. Slides were then counterstained with DAPI and coverslipped with Prolong Gold A.

### Image acquisition and analysis

Immunofluorescence images of the anterior corpus callosum were acquired using a Leica THUNDER computational confocal microscope. Regions of interest were first delineated according to the mouse brain atlas (Paxinos and Franklin 2008) at 5× magnification. Regions of interest were then imaged with 100× (oil) and 20× objectives and the 350-, 488-, 594-, or 647 nm channels. Images were taken in steps of 0.5 um (neuroanatomical experiments) or 0.22 um (RNAscope experiments) to subtend the entire focal plane of the immunofluorescence signal (12–24 steps per image). Stitched images were then imported into Imaris. All micrographs presented in the Results were taken from comparable regions across mice and experiments. For RNAscope experiments, a minimum of 2 sections per animal were used, where the entirety of the corpus callosum in both hemispheres was quantified. The expression of dopamine receptor d1 (*Drd1*) and dopamine receptor d2 (*Drd2*) mRNA by all cells within the region of interest expressing *Olig2* and/or *Pdgfra* mRNA was quantified semi-automatically using Object Detection and Nearest Distance features. Specifically, DAPI+ , *Olig2*+ , *Pdgfra*+ , *Drd1*+ , and *Drd2*+ cells were detected and then modeled through the creation of Surfaces using dedicated Imaris wizards. The Nearest Distance feature was then applied to compute distances between the Surfaces representing DAPI+ , *Olig2*+ , *Pdgfra*+ , *Drd1*+ , and *Drd2* + cells. The number of colocalizing structures (defined as zero–0.5 distance in µm) between these different Surface categories was then determined. All data was manually double checked and colocalizing structures misidentified by the automatic filter subtracted or added as necessary. Whenever there was doubt about whether a given cell expressed colocalization, it was not included in the analysis. All analyses were performed by 2 students, independently.

### Fast-scan cyclic voltammetry

Adult (> PND 90) male (*n* = 2) and female (*n* = 2) WT mice were killed via cervical dislocation and the brains removed quickly on ice before being transferred into ice-cold oxygenated HEPES-based buffer) in mM: 120 NaCl, 20 NaHCO_3_, 6.7 HEPES acid, 5KCL, 3.3 HEPES salt, 2 CaCl_2_, 2 MgSO_4_, 1.2 K_2_PO_4_ and 10 glucose) saturated with 95%O_2_/5% CO_2_. Three-hundred micrometer coronal slices containing the pregenum corpus callosum (+ 1.1 to + 1.4 mm anterior of Bregma; Franklin and Paxinos 2008) were obtained and left to recover at room temperature in HEPES-based buffer for at least 1 h prior to transferring to recording chamber and to aCSF (in mM: 124 NaCl, 62 NaHCo_3_, 3.8 KCl, 2.4 CaCl_2_, 1.3 MgSO_4_, 1.3 KH_2_PO_4_ and 10 glucose) saturated with 95% O_2_/5% CO_2_ for recording. Slices were warmed to 32 °C and carbon fiber microelectrode (CFM) inserted into non recording site for charging for 30 min prior to recording.

CFM were manufactured in-house using borosilicate glass (GC200F-10, Harvard Apparatus) and epoxy-free carbon fiber (7 μm diameter; tip length 50–100 μm; Goodfellow). Voltage waveform (− 0.7 V to + 1.3 V) was scanned at 8 Hz at 800 V/s across the recording CFM and switched out of circuit between scans using a Millar Voltammeter as described previously (Threlfell et al. 2010, 2012). Electrodes were calibrated using 1–2 μm dopamine in experimental medium. Calibration solutions were prepared immediately before calibration from a 2.5 mm stock solution in 0.1 m HClO_4_ stored at 4 °C. Signals were attributable to dopamine by the potentials for peak oxidation and reduction currents (oxidation peak: + 500–600 mV, reduction peak: ∼ − 200 mV).

### Electrical stimulation

Recordings were obtained from the corpus callosum and mediodorsal striatum. The electrodes were moved in series so that stimulation and recording occurred first in the corpus callosum. The recording electrode was then moved to the nearest part of the adjacent striatum, and after stimulating the corpus callosum and recording from the striatum, the stimulating electrode was then moved to the striatum. Dopamine release was evoked by a local bipolar concentric Pt/Ir electrode (25 μm diameter; FHC Inc., Bowdoin, ME, USA) placed approximately 100 μm from the CFM. Stimulus pulses (200 μs duration) were given at 0.6 mA. Dopamine neurons in vivo exhibit a range of firing frequencies from ∼1–40 Hz or higher. We applied either single pulses (1p) or 20 pulses (20p) at 20 Hz. Mean peak [DA]_o_ evoked by 1p was equivalent to that of a 1 Hz train; 1p is used in frequency comparison to indicate maximum 1 Hz data.

Electrical stimulations were repeated at 2.5 min intervals, which allow stable release to be sustained over several hours. Each stimulus type was repeated in triplicate in a random order. These repeats were then averaged within an experiment and normalized and averaged across different animals.

### Statistical analysis

RNAscope and fast scan cyclic voltammetry (FSCV) data are expressed as means ± SEM and the sample size, *n* = technical repeats and *N* = number of animals. Quantitative differences in colocalization of dopamine receptor transcripts by oligodendrocyte lineage cells was analyzed using a 2 (oligodendrocyte lineage cell type) × 2 (dopamine receptor subtype) mixed ANOVA. Comparisons for differences in FSCV means were made using Mann–Whitney tests. All analyses were performed in GraphPad Prism 9.0.

## Results

### Midbrain-originating dopamine axons laden with varicosities are present in abundance in the anterior corpus callosum

**Fig. 1:**
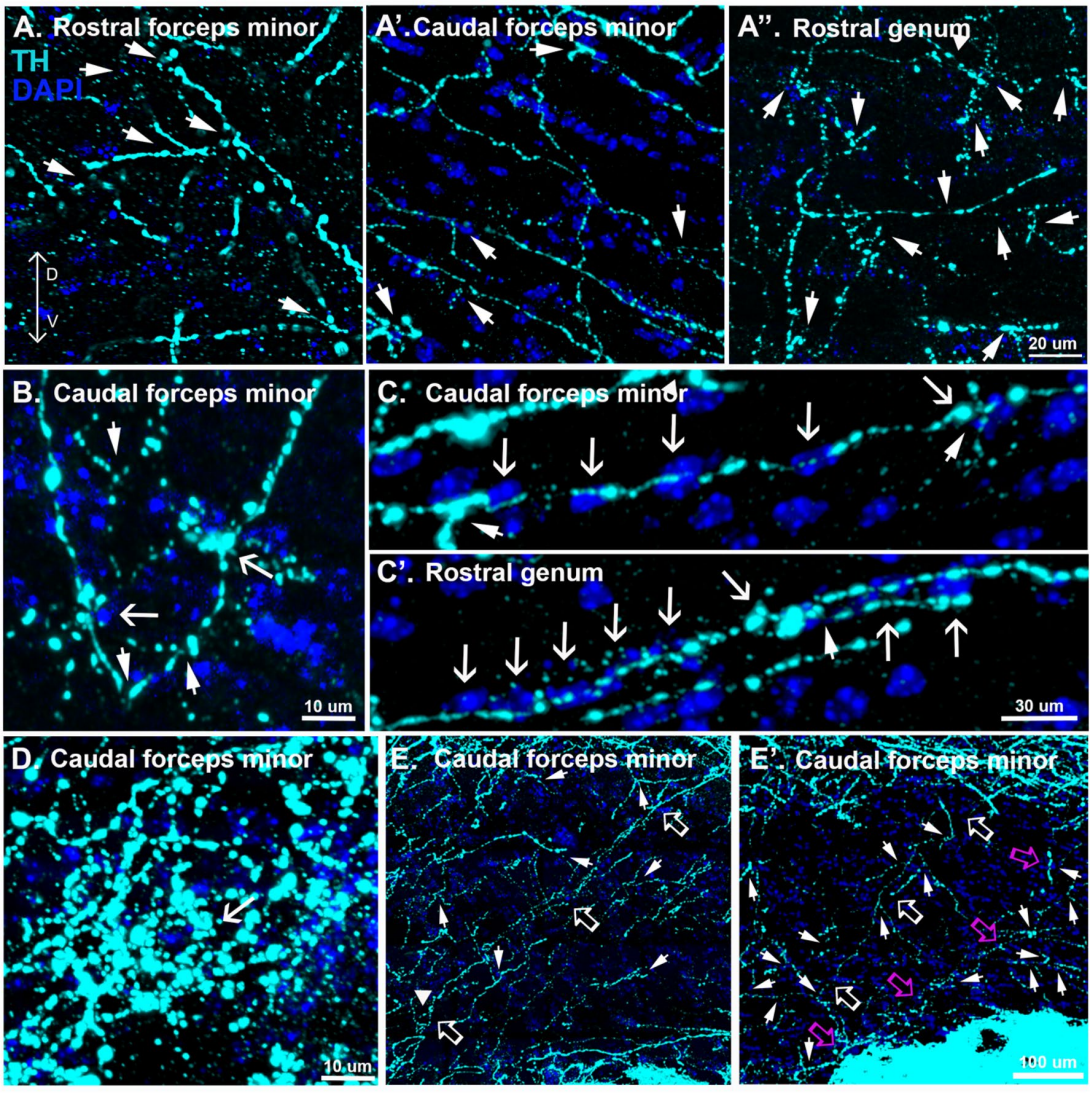
Characteristics of corpus callosal TH+ axons. **A**–**A”** Computational confocal sections indicate that TH+ axons do not demonstrate a clear topographical organization (arrowheads indicate branching sites). **B** TH+ axons make perisomatic terminations (arrowheads indicate branching sites, arrows indicate perisomatic terminations). **C**, **C’** TH+ axons align with rows of nuclei (arrowheads indicate branching sites, arrows indicate nuclei). **D** Multiple TH+ axons converge onto single cells (arrow). **E**, **E’** TH+ axons adjoin striatal and cortical regions across the dorso-ventral axis (different axons identified by arrows of different colors, arrowheads indicate branching sites)

If midbrain dopamine neurons play a role in myelin plasticity of the corpus callosum, their axons should be present in this white matter tract. In coronal sections from adult mice, tyrosine hydroxylase (TH) immunoreactivity, indicative of monoaminergic innervation, is typically observed most readily in the striatum and prefrontal cortex. However, we found that increasing the exposure, which saturated the signal in the nearby striatum and prefrontal cortex, revealed abundant TH+ axons, including associated varicosities throughout the anterior corpus callosum, albeit at a much lower density than the neighboring striatum and prefrontal cortex (Fig. 1, Fig S1). The pattern of innervation was patchy, with areas of higher density neighbored by small areas nearly devoid of TH+ axons (Fig S1 insets). The highest density of TH+ axons was in the medial aspect of the caudal forceps minor, the rostral genu and in the external capsule (Fig S1).

**Fig. 2:**
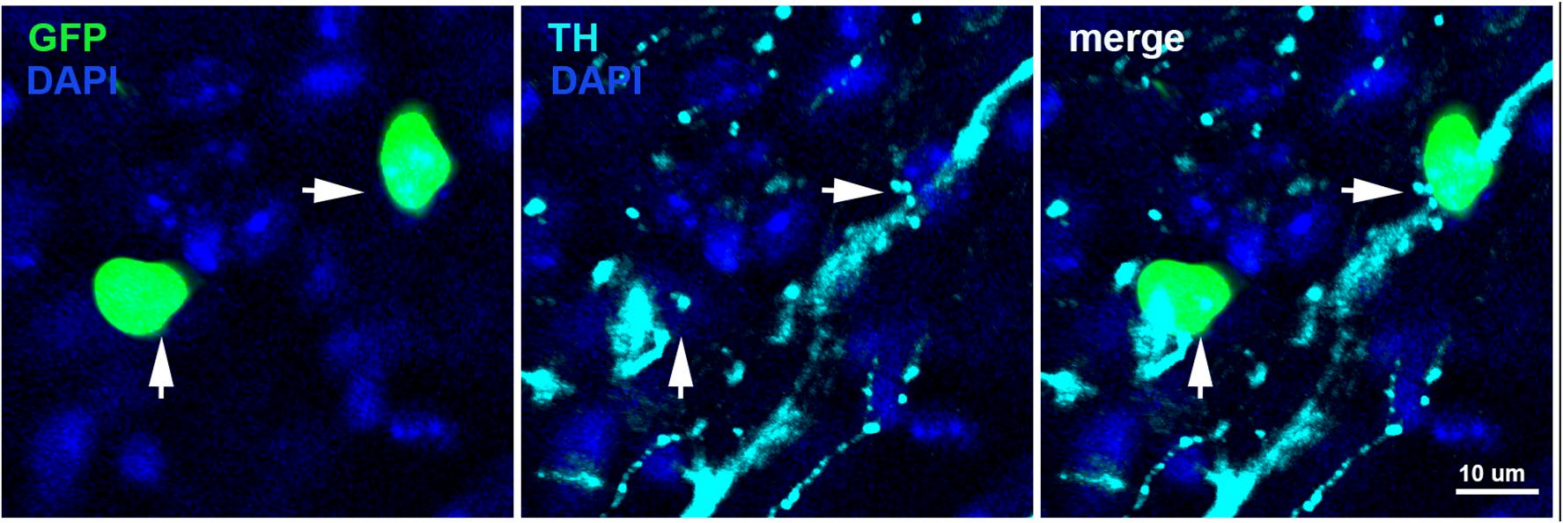
OPCs receive perisomatic inputs from TH+ axons in the anterior corpus callosum. TH+ axons make perisomatic terminations (arrowheads) with GFP + nuclei in PDGFRa-GFP reporter mice

TH+ axons in the anterior corpus callosum lacked topographical organization. Rather, in all subregions examined, some TH+ axons were observed coursing dorsoventrally while others coursed along the mediolateral axis (Fig. 1A–B, Fig S2). In many instances, TH+ axons exhibited branching (Fig. 1A–C, E, arrowheads, Fig S2). At higher magnifications, TH+ axons in the corpus callosum exhibited varicosities (Fig. 1B–D, arrowheads, Fig S3) and many of these varicosities surrounded perisomatic sites (Fig. 1B, arrows, Fig S3) or followed a trajectory that aligned with rows of nuclei (Fig. 1C, arrows, Fig S4). There were also instances where what appear to be several independent TH+ axons converge onto individual cells (Fig. 1D, Fig S5). In a separate series of experiments conducted in PDGFRa reporter mice, we identified OPCs as one of the cell populations upon which TH+ axons make perisomatic terminations (Fig. 2, arrowheads). Furthermore, in the caudal forceps minor of the corpus callosum, branching TH+ axons in the dorsoventral orientation were occasionally observed adjoining striatal and cortical regions (Fig. 1E, arrows).

**Fig. 3:**
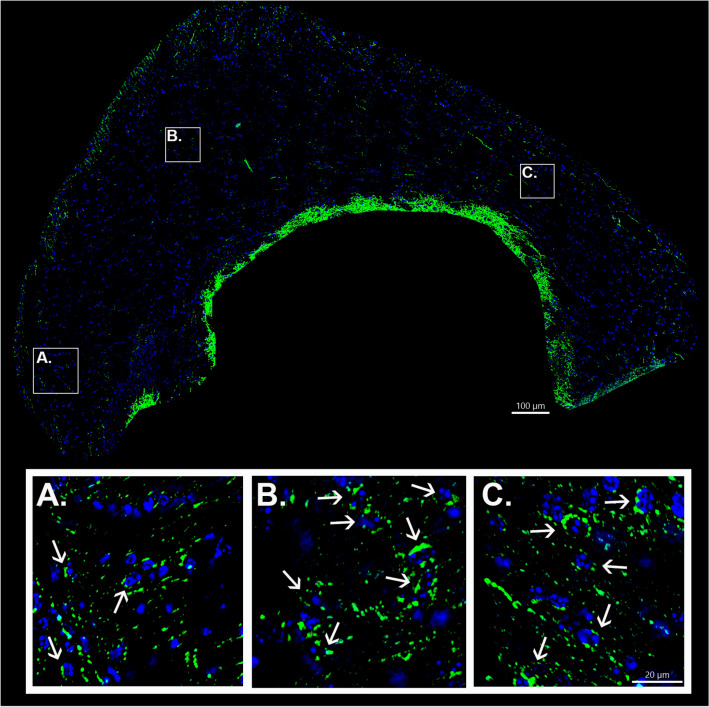
Computational confocal images demonstrate eYFP+ axons in the caudal forceps minor of the corpus callosum and external capsule of adult male DAT^cre^ mice. Sources of high magnification examples are indicated by white bounding boxes (**A**–**C**). Note the lack of topographical organization and the frequent occurrence of perisomatic terminations

The above results suggest that monoaminergic axons innervate the anterior corpus callosum and terminate on cells within its regions. However, TH immunoreactivity can be indicative of either dopamine or norepinephrine terminals. To determine if TH+ axons observed in the corpus callosum originated from midbrain dopamine neurons, a viral Cre-lox strategy was employed. To selectively label dopamine axons with eYFP, adult male DAT-Cre mice (*n* = 3) were stereotaxically injected with a Cre-dependent eYFP reporter virus into the midbrain unilaterally as part of previously published study (Reynolds et al. 2019). eYFP+ axons in the corpus callosum were detected in alternate sections by immunostaining. As was the case with TH, eYFP+ axons could be observed throughout the rostral and caudal aspects of the anterior corpus callosum, albeit at a seemingly lower density than TH+ axons (Fig. 3, Figs S6, S7). Similar to TH+ corpus callosum axons, eYFP+ axons lacked topographical organization, with some axons coursing dorsoventrally and others mediolaterally (Fig. 3, Figs S6, S7 insets). eYFP+ axons also appeared beaded with frequent occurrences of perisomatic terminations (Fig. 3, arrows). Furthermore, branches of eYFP+ axons could be observed invaginating themselves between adjoining nuclei (Fig S6, arrowheads). eYFP+ axons could also be observed coursing into (or out of) the corpus callosum via striatal and cortical regions (Fig S7, arrows) in a pattern similar to that observed for TH+ axons. Thus, TH+ axons observed in the corpus callosum could, at least in part, be attributable to dopaminergic innervation.

### Dopamine-like signals can be detected following local electrical stimulation in the anterior corpus callosum

**Fig. 4:**
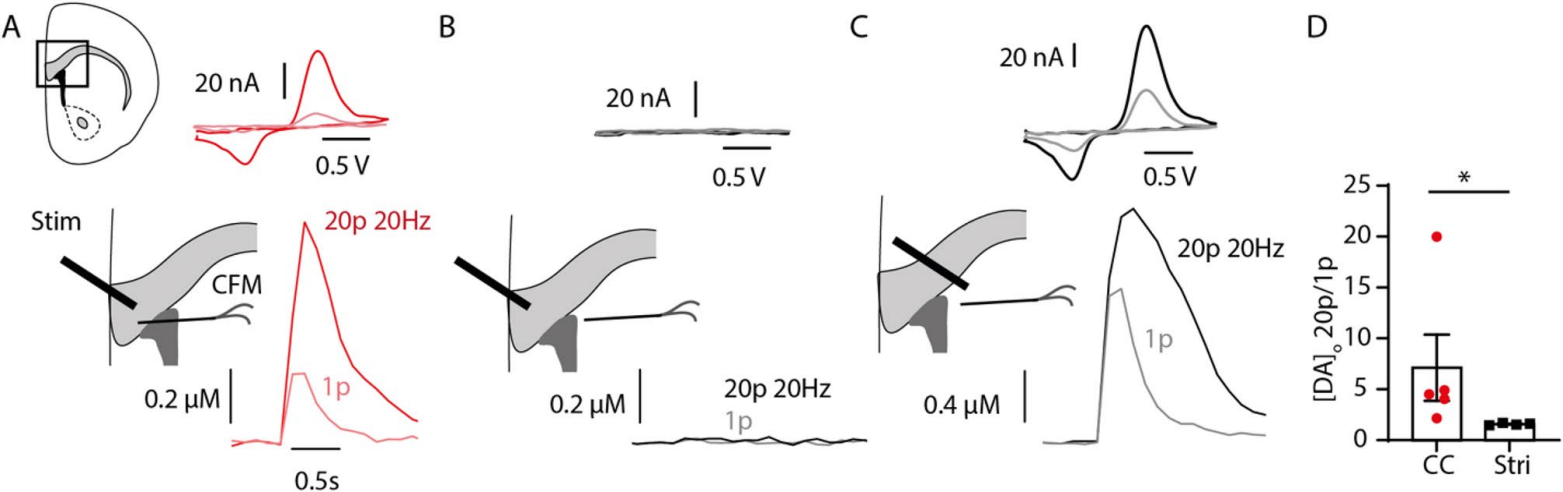
Fast-scan voltammetry reveals dopamine-like signals in the anterior corpus callosum. **A**–**C** Left panel: Cartoon showing stimulating electrode and carbon-fiber microelectrode (CFM) placement in the corpus callosum (**A**), corpus callosum/striatum (**B**) and striatum (**C**); **A**–**C** center panel: example traces of [DA]_o_ vs time evoked by 1p (light color) or 20p 20 Hz (dark color). **A**–**C** top panel: cyclic voltammograms showing oxidation (~ 620 mV) and reduction (~ 20 mV) peaks consistent with dopamine in the corpus callosum (**A**) and striatum (**C**), from the peak of the transient. (**D**) Summary of the ratio of peak [DA]_o_ following 20p 20 Hz:1p *n* = 5, 4 sites from 2 animals

To determine if dopamine is released by axons in the corpus callosum, we used local electrical stimulation and FSCV at CFM. FSCV is a well-established method for measuring evoked release of monoaminergic neurotransmission (Patel and Rice 2013). It works by measuring the oxidation and reduction potentials of neurotransmitter species in response to a time-dependent electrical stimulation, such that a time-locked oxidation response can be measured. When both the stimulating and recording electrode were placed within the corpus callosum we recorded electrochemical monoamine signals above our detection threshold in ~ 40% of recorded locations, consistent with local release of dopamine from patchy axonal innervation (Fig. 4A, Fig S8). We then used two key strategies to confirm that these dopamine-like signals were indeed released locally and were not due to diffusion of dopamine from the nearby striatum.

First, we tested whether the signals detected in the corpus callosum were due to current spread to the nearby striatum which in turn may have evoked release of striatal dopamine, leading to its diffusion to the corpus callosum. When we subsequently moved the recording electrode to the nearby striatum, we could not detect any monoamine signals in response to stimulation of the corpus callosum (Fig. 4B). However, when we also moved the stimulation electrode to the striatum, this same stimulation protocol successfully evoked striatal dopamine release (Fig. 4C).

Second, we compared the ratio of peak monoamine release in the corpus callosum and striatum by two different local stimulation protocols (20p 20 Hz versus 1p). As expected for electrically evoked dopamine release under drug-free conditions in the striatum (Brimblecomb et al. 2018; Rice and Cragg 2004) where local release of acetylcholine acts on axonal nicotinic acetylcholine receptors to powerfully regulate striatal dopamine release, this 20p:1 ratio was low (Fig. 4D). In contrast, the ratio in the corpus callosum was significantly higher and more variable than in striatum (Mann-Witney *P* = 0.016, Fig. 4D), further indicating that the putative dopamine signal in the corpus callosum is distinct from the locally evoked striatal dopamine signal.

### Oligodendrocyte lineage cells in the anterior corpus callosum express dopamine receptor transcripts

**Fig. 5:**
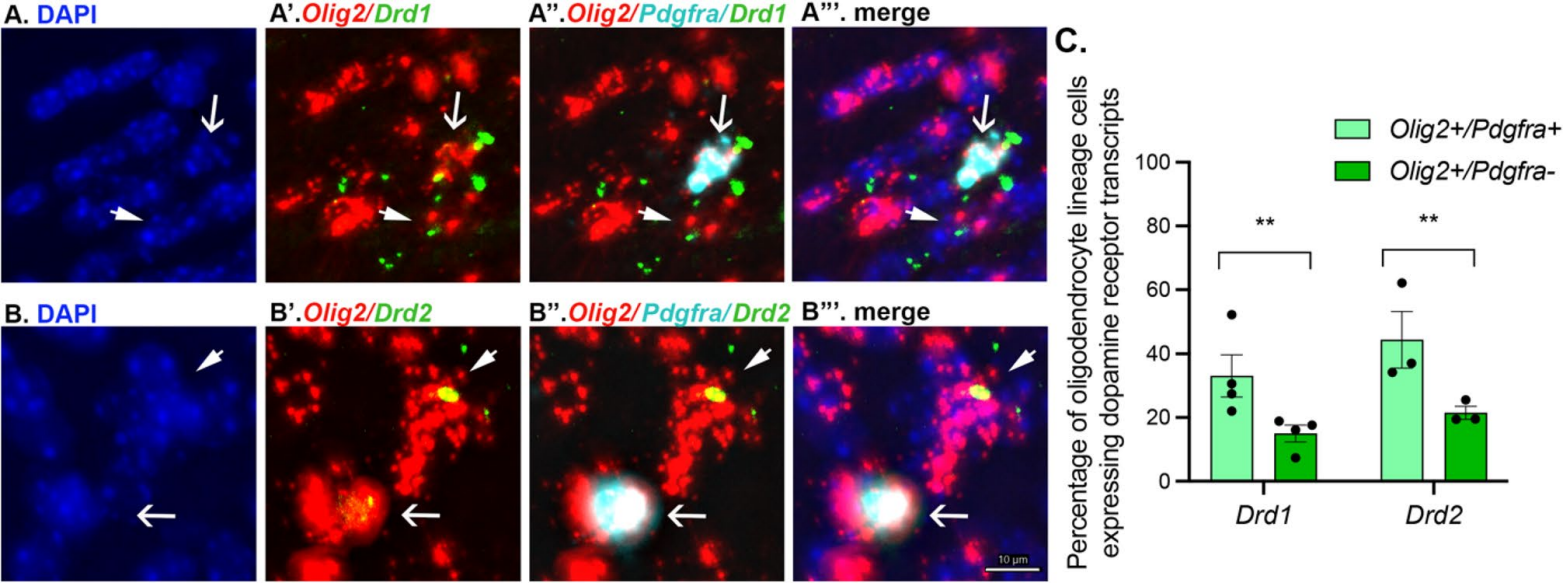
Oligodendrocyte lineage cells express dopamine receptor 1 (*Drd1*) and dopamine receptor 2 (*Drd2*) transcripts in anterior regions of the corpus callosum. Computational confocal images demonstrating DAPI (**A**, **B**); *Olig2* and *Drd1* (**A’**) or *Olig2* and *Drd2* (**B’**) RNA probes; *Olig2*, *Pdgfra* and *Drd1* (**A’’**) or *Olig2*, *Pdgfra* and *Drd2* (**B’’**) RNA probes; and the merge of all channels (**A’’’**, **B’’’**). Arrows indicate *Olig2* + /*Pdgfra* + cells that colocalize with dopamine receptor transcripts. Arrowheads indicate *Olig2* + /*Pdgfra-* cells that colocalize with dopamine receptor transcripts. **C** Quantification of dopamine receptor transcripts by oligodendrocyte lineage cells in the anterior corpus callosum. On average, there are nearly twofold more *Olig2* + /*Pdgfra* + cells than *Olig2* + /*Pdgfra-* expressing dopamine receptor transcripts (*F*_(1, 11)_ = 13.9, **p < .01). Each black dot represents the average of 2–4 brains sections per animal

We next examined if oligodendrocyte lineage cells in the anterior corpus callosum express *Drd1* or *Drd2* transcripts with RNAscope. Using coronal sections obtained from adult male and female wild-type mice containing the forceps minor or rostral genu of the corpus callosum, we observed that *Drd1* and *Drd2* transcripts are expressed by ~ 40% of *Olig2* + */Pdgfra* + cells (Fig. 5, arrows) and ~ 20% of *Olig2* + */Pdgfra-* cells (Fig. 5, arrowheads), suggesting higher levels of *Drd1* and *Drd2* transcripts in OPCs that decline as these cells differentiate into oligodendrocytes. Consistent with previous studies that conducted whole brain single cell RNA sequencing or using mPFC brain tissue (Marques et al. 2016; Zhang et al. 2014), the number of transcripts expressed by oligodendrocyte lineage cells in the corpus callosum is noticeably less compared to non-oligodendrocyte lineage cells in the mPFC and in the striatum (Fig S9). However, oligodendrocyte lineage cells in these regions also express *Drd1* and *Drd2* transcripts at levels similar to those observed in the corpus callosum (Fig S9).

## Discussion

The corpus callosum is the largest white matter tract in the brain, responsible for coordinating cortical regions in the left and right hemispheres by carrying contralaterally projecting cortical axons. Myelination within this pathway is vital to the degree of coordination across hemispheres, as evidenced by developmental and disease states which impact the corpus callosum, such as schizophrenia (Karlsgodt 2016; Koshiyama et al. 2018; Lee et al. 2013; Mighdoll et al. 2015; Rotarska-Jagiela et al. 2008; Samartzis et al. 2014; Tamnes and Agartz 2016; Xu and Li 2011). Recent evidence has demonstrated that typical adult myelination is much more dynamic than previously thought (Knowles et al. 2022), raising the possibility for experience and external factors to modulate interhemispheric coordination. Here we provide the first evidence we know of that midbrain dopaminergic neurons are situated for regulation of corpus callosum myelination. Moreover, the regions of the anterior corpus callosum we demonstrate to have abundant dopamine axons have been reported to be most altered in disease states such as schizophrenia (Koshiyama et al. 2018; Lee et al. 2013; Rotarska-Jagiela et al. 2008).

We observed a large number of dopaminergic fibers originating from the midbrain in anterior regions of the corpus callosum, specifically the forceps minor and rostral genu, as well as in the neighboring external capsule. These axons show branching and perisomatic varicosities, consistent with terminations. Electrical stimulation of the corpus callosum produces an increase in dopamine-like signals, indicating the presence of functional release sites in this region. While FSCV cannot distinguish between dopamine and norepinephrine signals, the CFMs used in our experiments have a twofold greater sensitivity for the former than for the latter (Park et al. 2011). Given the possibility that norepinephrine axons innervate the anterior corpus callosum, as has previously been reported for the cingulum (Jones and Moore 1977), it is likely that the FSCV signal recorded here is not exclusively dopamine. Yet, together with the appearance of perisomatic dopamine fiber terminations, the likelihood of functional dopamine signal in this region is plausible. Finally, we also show that oligodendrocyte lineage cells in this region express *Drd1* and *Drd2* transcripts, indicating the potential for dopamine regulation of these cells.

Environmental stimuli dynamically regulate proliferation and differentiation of OPCs, myelin production by mature oligodendrocytes, as well as the geometry of existing myelin sheaths in the adult corpus callosum (Bacmeister et al. 2020; Geraghty et al. 2019; Gibson et al. 2014; Kato et al. 2020; Lehmann et al. 2017; Liu et al. 2012, 2016; Makinodan et al. 2012; McKenzie et al. 2014; Marques et al. 2016; Mitew et al. 2018; Osso et al. 2021; Pan et al. 2020; Piscopo et al. 2018; Sampaio-Baptista et al. 2020; Steadman et al. 2020; Xiao et al. 2016; Yang et al. 2020; Zheng et al. 2019). These phenomena, examples of myelin plasticity, are required for experiential-induced behavioral plasticity (Liu et al. 2016; McKenzie et al. 2014; Pan et al. 2020; Steadman et al. 2020; Xiao et al. 2016) and are thought to modulate brain function by fine-tuning the synchrony of activity across multiple neural circuits that vary in distances from one another (Etxebarria et al. 2016; Noori et al. 2020; Pajevic et al. 2014), although other mechanisms may also be at play (for review see Xin and Chan 2020). Our finding of midbrain dopamine axons in the corpus callosum suggests dopamine neurotransmission may play a role in myelin plasticity of this white matter tract.

A question that arises from our results relates to the sites of origin and characteristics of the dopamine neurons that give rise to the axons in the corpus callosum. Mesolimbic, mesocortical and mesostriatal dopamine pathways are widely regarded as each having a single regional target, such that, for example, mesolimbic neurons do not also innervate cortical regions (Yetnikoff et al. 2014). It is unclear from the present study how dopamine white matter axons fit within this taxonomy. Upon close examination of micrographs and tracing plots, it can be seen that while some dopamine axons invaginate the anterior corpus callosum via striatal and cortical regions (see Figs S1 and S7), the afferent trajectories of other dopamine axons are not discernable. It is also evident that some dopamine axons cross the corpus callosum along the dorsoventral axis to adjoin striatal and cortical regions (see Fig. 1). Further work is needed to understand how white matter dopamine axons fit within the known trajectories of midbrain dopamine pathways. This question will be addressed by injecting into the anterior corpus callosum of DAT^cre^ mice a cre-dependent retrograde AAV virus.

In regard to the characteristics of the afferent dopamine neurons, it has been increasingly recognized that midbrain dopamine neurons are genetically, anatomically, and functionally heterogeneous (Anderegg et al. 2015; de Jong et al. 2022; Poulin et al. 2014, 2018). Recent work has classified these cells into seven distinct subtypes based on the unique combinations of characteristics they display (Poulin et al. 2020. As one example, some midbrain dopamine neurons are capable of glutamate co-transmission (Eskenazi et al. 2021), which is particularly intriguing in light of the fact that glutamate can promote myelination (Habermacher et al. 2019). Midbrain dopamine-glutamate neurons may therefore be uniquely positioned to contribute to myelin plasticity via bifunctional mechanisms. Whereas atypical antipsychotics can increase proliferation and differentiation of OPCs (Bongarzone et al. 1998; Howard et al. 1998; Mi et al. 2018; Rosin et al. 2005; Wang et al. 2010), AMPA-mediated glutamate neurotransmission reduces OPC proliferation and promotes OPC differentiation (Fannon et al. 2015; Gallo et al. 1996; Gudz et al. 2006; Liu and Almazan 1995; Mangin et al. 2012; Yuan et al. 1998). Although we cannot determine from the present work the specific characteristics of the midbrain dopamine neurons providing afferents to the corpus callosum, it is possible that midbrain dopamine afferents include dopamine-glutamate neurons.

A role for dopamine in myelin plasticity of the corpus callosum necessitates not only the presence of dopamine axons, but also the expression of dopamine receptors by oligodendrocyte lineage cells in these regions. Consistent with the knowledge that OPCs receive synaptic and extra-synaptic neuronal inputs (Kula et al. 2019) and are regulated by atypical antipsychotics (Bongarzone et al. 1998; Howard et al. 1998; Mi et al. 2018; Rosin et al. 2005; Wang et al. 2010), our experiments demonstrated that OPCs in the anterior corpus callosum express *Drd1* and *Drd2* transcripts, albeit at substantially lower levels than in striatal or mPFC neurons (see Fig S9). While the protein expression levels of these receptors by OPCs remain to be established, our data support the possibility that these cells could respond to midbrain dopamine neuron activity through changes in cell proliferation, differentiation, and/or even non-myelin related functions (Xin and Chan 2020), possibly via dopamine-stimulated changes in intracellular calcium mobilization (Undieh 2010). It is not known from the present data whether the same proportion of OPCs expressing dopamine receptor transcripts are also abutted by dopamine terminals; however, this may not be relevant to the potential effects of dopamine on OPC activity, as dopamine could act upon these cells via both synaptic and extrasynaptic mechanisms (i.e., volume transmission; Velez-Fort et al. 2010; Fuxe et al. 2015; Borroto-Escuela et al. 2018; Kula et al. 2019; Wienche et al. 2020).

We also found that *Olig2*+ */Pdgfra-* cells in the anterior corpus callosum also express *Drd1* and *Drd2* transcripts. While we cannot determine from the present study whether these cells are premyelinating oligodendrocytes or mature oligodendrocytes, this result is nonetheless surprising because upon differentiation from OPCs, oligodendroglia lose their synapses and no longer display post-synaptic potentials in response to neural activity (Butt et al. 2014). One possible explanation for this finding is that the *Olig2*+ */Pdgfra-* cells expressing dopamine receptor transcripts are recently differentiated or are in the midst of transitioning into maturity and have not completed the process of synapse elimination. However, it is important to consider that dopamine signaling in *Olig2*+ */Pdgfra-* cells may serve functions other than myelin plasticity, such as providing metabolic support or mediating neuro-immune responses (Xin and Chan 2020). It is important to keep in mind that because dopamine D2 receptors are also expressed by dopamine axons as autoreceptors, we cannot exclude from these experiments the possibility that *Drd2* transcripts may also be expressed in the corpus callosum by dopamine axons impinging on *Olig2*+ */Pdgfra*+ and *Olig2*+ */Pdgfra-* cells.

In summary, we demonstrate here that the anterior corpus callosum contains functional midbrain dopamine axons and that oligodendrocyte lineage cells in this white matter tract express dopamine receptor transcripts. These data implicate midbrain dopamine neurons in myelin regulation and plasticity, although the precise function of dopamine-oligodendroglia communication remains to be established. It has previously been postulated that because midbrain dopamine neurons are targeted by a brain-wide network of afferent inputs and have broad efferent projections (Yetnikoff et al. 2014), they are in a unique position to play a key role in the synchronization of parallel information streams (Beeler and Dreyer 2019). Perhaps one mechanism by which they may contribute to information synchronization is through the regulation of myelin plasticity.

### Supplementary Information

Below is the link to the electronic supplementary material.

Supplementary file1 (DOCX 10554 KB)

## Data Availability

The datasets generated and/or analyzed during the current study are available upon reasonable request.
