# Supplementary material for: Axo-glial interactions between midbrain dopamine neurons and oligodendrocyte lineage cells in the anterior corpus callosum
Source: Brain Struct Funct. 2023 Sep 5;228(8):1993–2006. doi: 10.1007/s00429-023-02695-y (PMC10516790; doi:10.1007/s00429-023-02695-y)
Supplement: Supplementary file 1 — Supplementary file1 (DOCX 10554 KB) [file 429_2023_2695_MOESM1_ESM.docx]

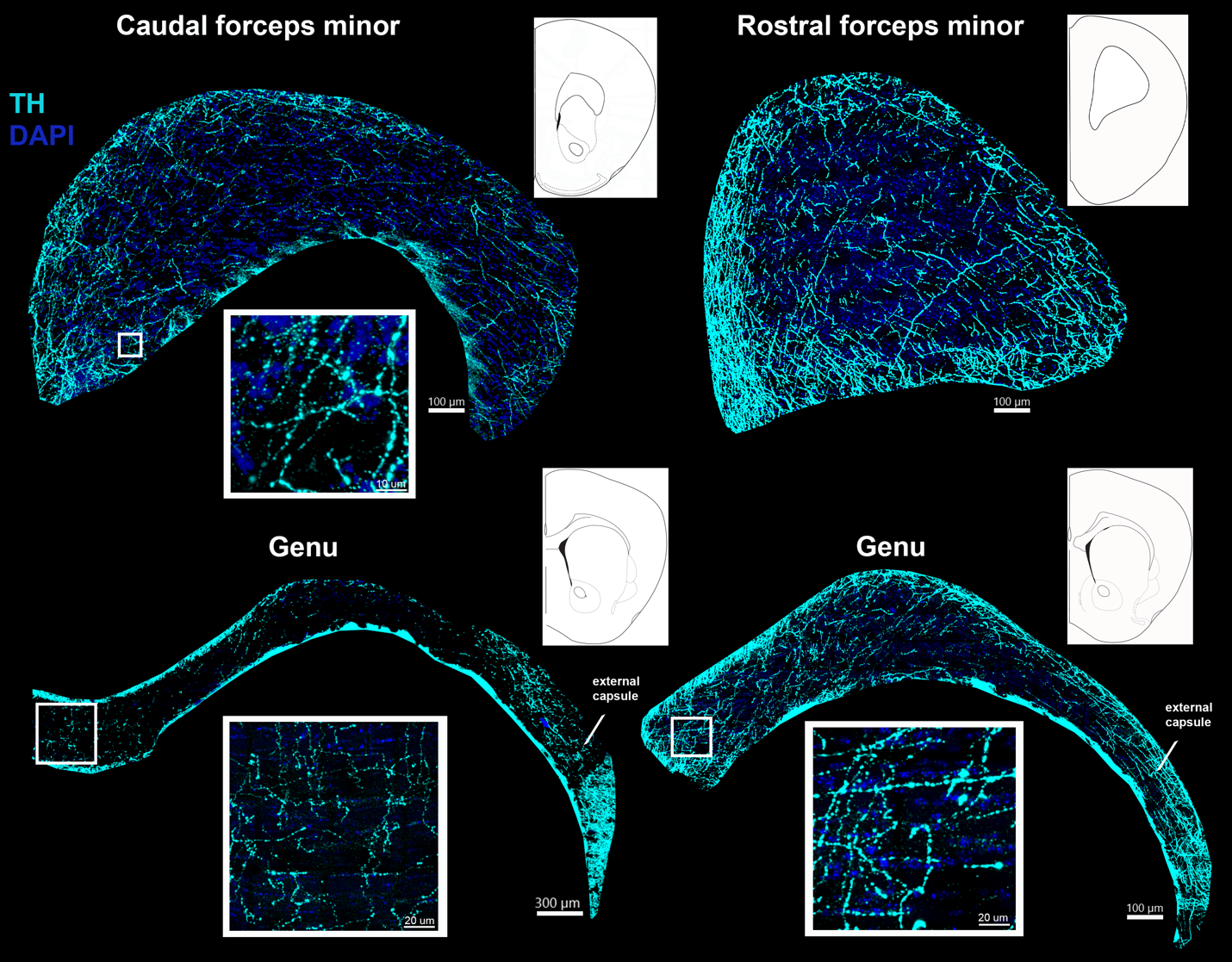


**Fig. S1** TH+ axons are present in all levels of the forebrain corpus callosum. Top row: TH+ axons are present in the most rostral regions of the corpus callosum, the forceps minor. Bottom row: TH+ positive axons in the genu of the corpus callosum and the external capsule. The sources of high magnification examples are indicated by the white boxes. Schematics to the right of each example identifies the location along the rostral-caudal axis. Note the lack of topography in the organization of TH+ axons and the higher density of TH+ axons in the medial aspects of the caudal forceps minor (top row, left), rostral genu (bottom row, right) and external capsule (bottom row).


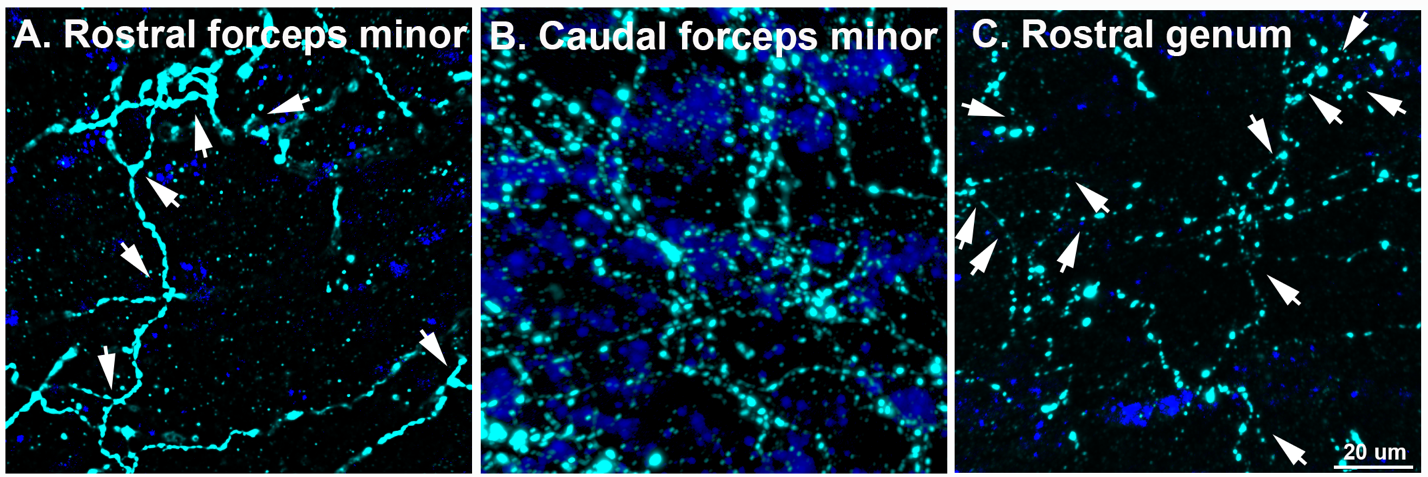


**Fig. S2** TH+ axons in the anterior corpus callosum lack topographical organization and demonstrate branching (arrowheads indicate branching sites).


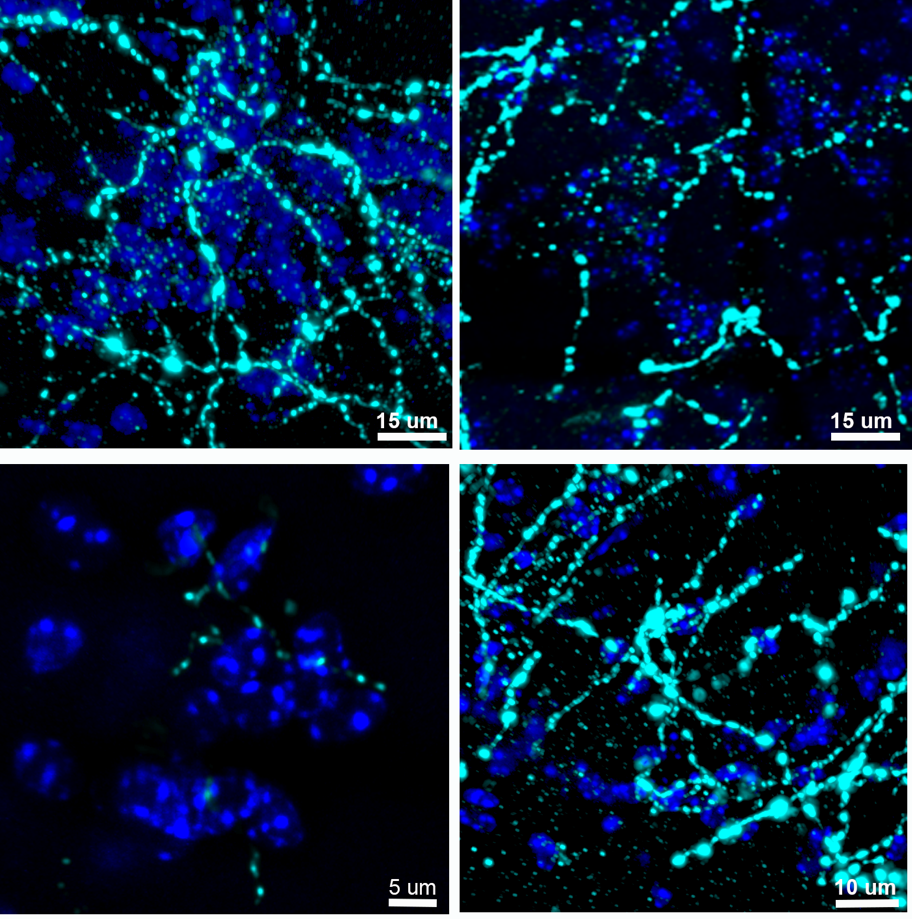


**Fig. S3** TH+ axons in the anterior corpus callosum demonstrate varicosities and perisomatic terminations. Note the interstitial organization of TH+ axons and how they frequently wrap around individual cell nuclei. The scale bar in each row applies to all images in that row.


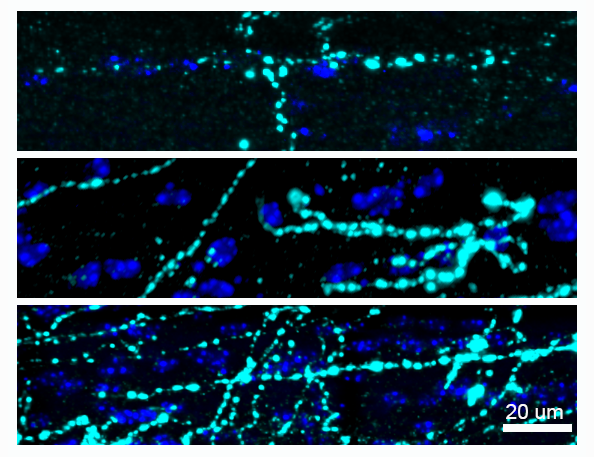


**Fig. S4** TH+ axons were often observed juxtaposed along rows of cell nuclei in caudal forceps minor and rostral genu regions of the corpus callosum.


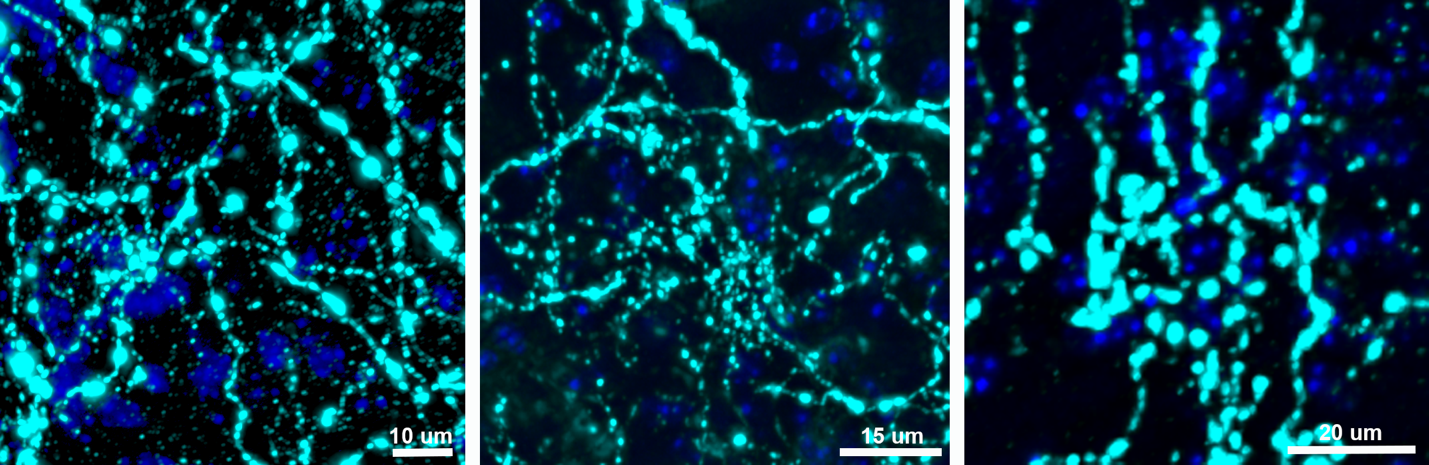


**Fig. S5** Individual cells in the forebrain corpus callosum are frequently impinged upon by converging TH+ axons. Examples were obtained from regions in the caudal forceps minor or rostral genu of the corpus callosum.


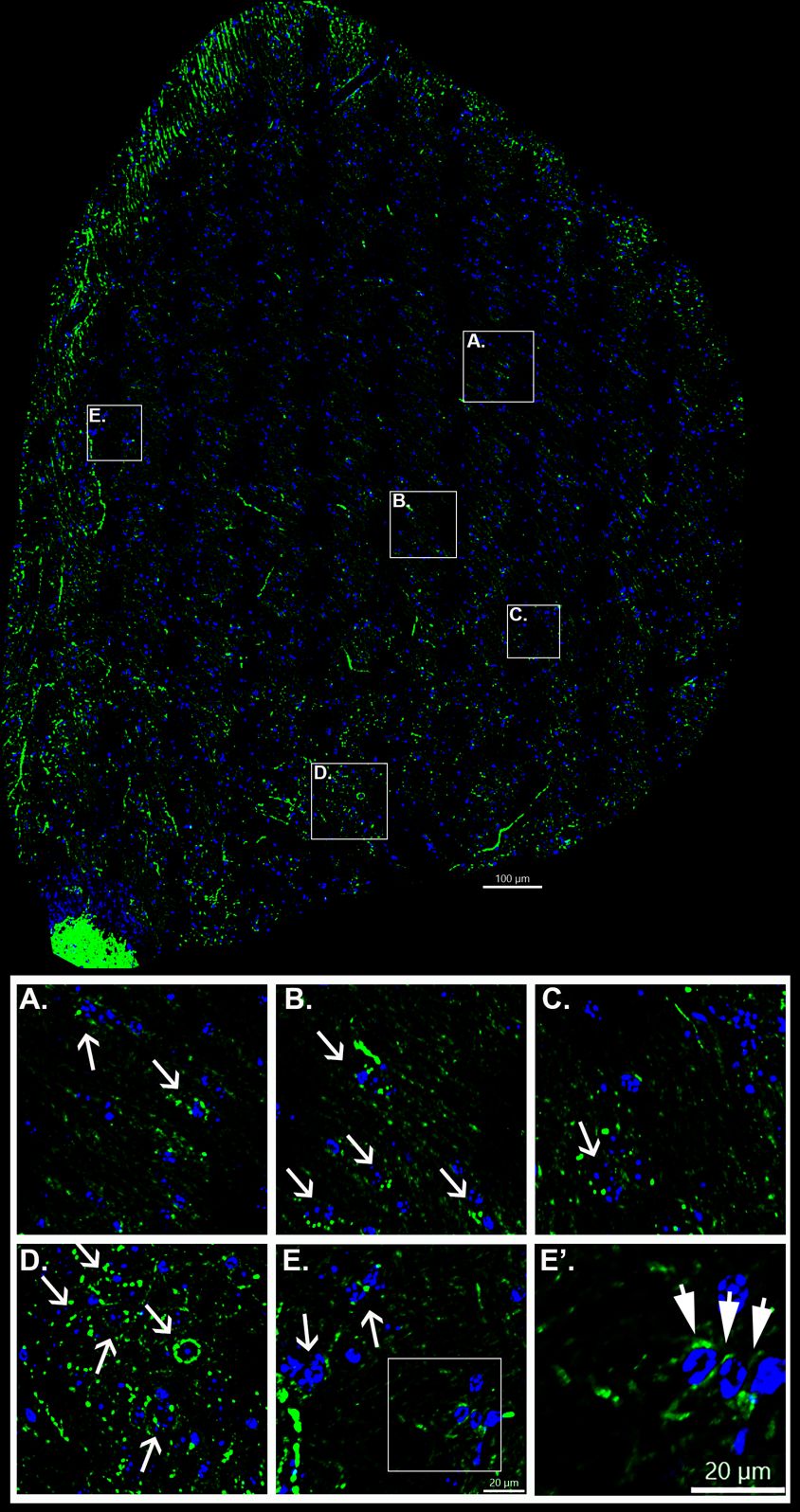


**Fig. S6** eYFP+ axons in the rostral forceps minor of the corpus callosum of adult male DAT^cre^ mice. Note the high density of eYFP+ axons in the rostral pole of the nucleus accumbens. Sources of high magnification examples are indicated by white bounding boxes (A – E). Note the lack of topographical organization and the frequent occurrence of perisomatic terminations. E’ is a high magnification of the region identified by the white box in E. Note the axon branching and interstitial organization.


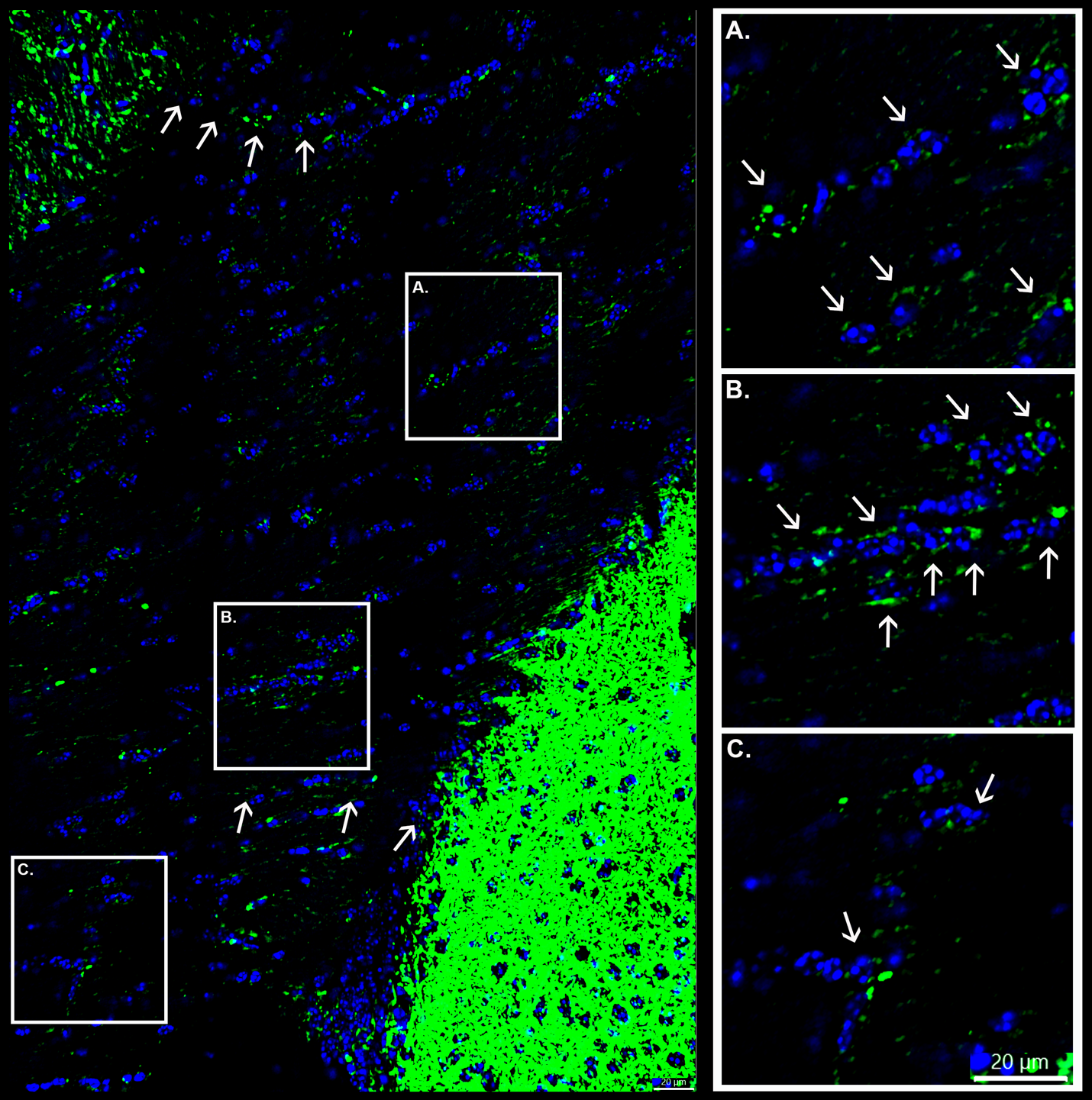


**Fig. S7** eYFP+ axons in the rostral genu of the corpus callosum of adult male DAT^cre^ mice. Sources of high magnification examples are indicated by white bounding boxes (A – C). Note the frequent occurrence of perisomatic terminations. Note also how individual eYFP+ axons can be observed leaving/entering the corpus callosum from striatal and cortical regions (arrows).


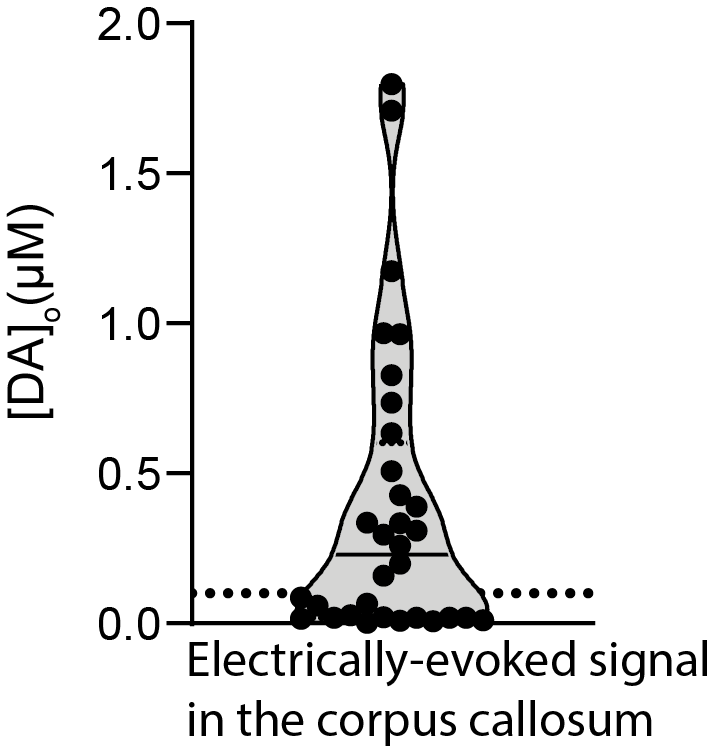


**Fig. S8** Summary of the ratio of peak [DA]_o_ following 20p 20Hz:1p in the corpus callosum of drug free wild-type mice (n = 32, N = 2M, 2F). Dotted line shows region below detection threshold; 18/32 recordings are above detection, corresponding to 6/20 recording sites.


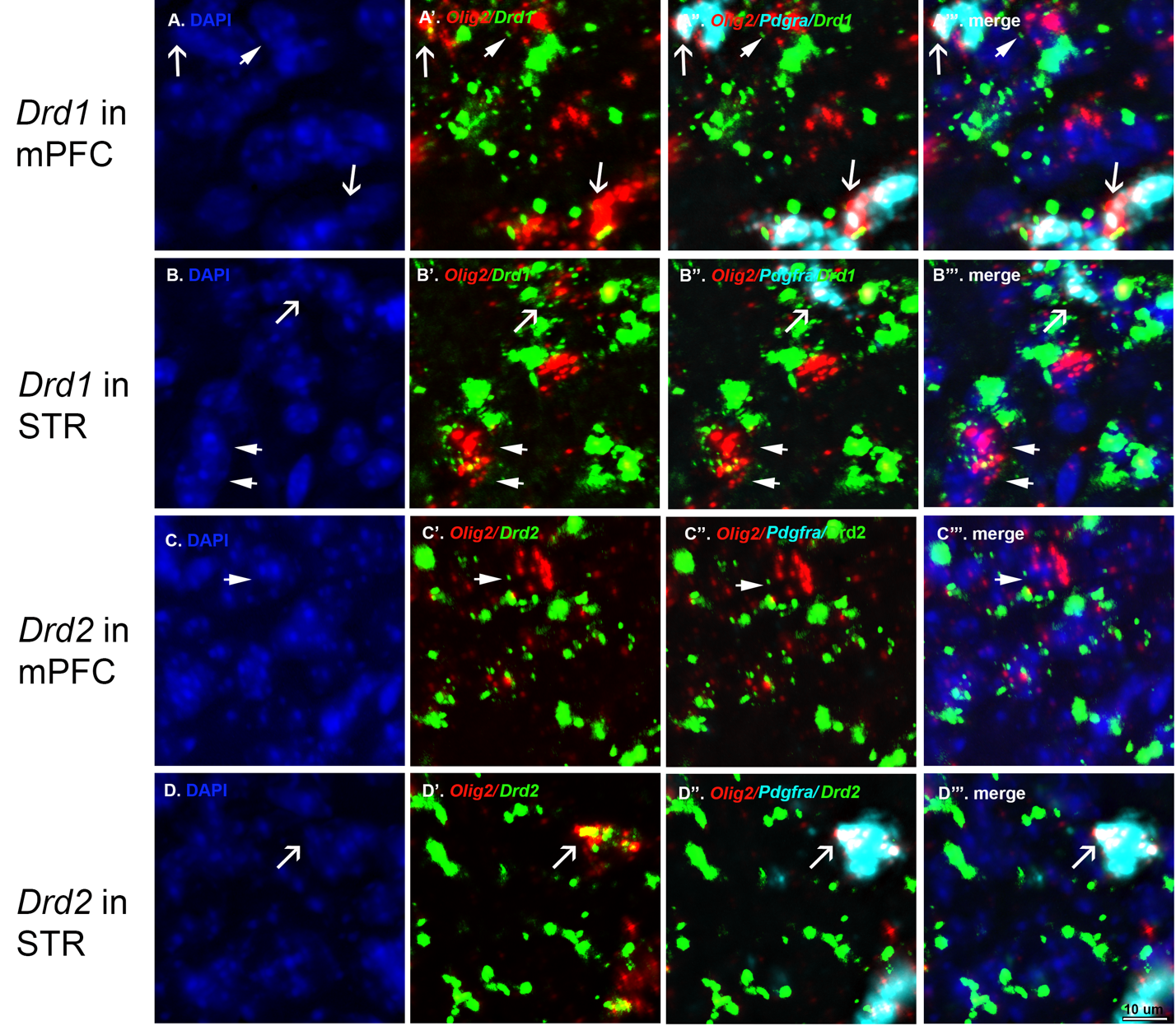


**Fig. S9** Expression of *Drd1* and *Drd2* receptor transcripts in the medial prefrontal cortex and striatum. Computational confocal images demonstrating DAPI (A - D); *Olig2* and *Drd1* (A’, B’) or *Olig2* and *Drd2* (C’, D’) RNA probes; *Olig2*, *Pdgfra* and *Drd1* (A’’, B’’) or *Olig2*, *Pdgfra* and *Drd2* (C’’, D’’) RNA probes; and the merge of all channels (A’’’ – D’’’). Note the difference in level of *Drd1* and *Drd2* transcript expression compared to the corpus callosum (Fig 4, obtained from same images). Intriguingly, some *Olig2+*/*Pdgfra+* cells (arrows) and *Olig2+*/*Pdgfra-* cells (arrowheads) in the mPFC and striatum also express *Drd1* and *Drd2* transcripts.
